# Inter-agency collaboration factors affecting multidisciplinary workers’ ability to identify child maltreatment

**DOI:** 10.1186/s13104-020-05162-7

**Published:** 2020-07-06

**Authors:** Ayumi Okato, Tasuku Hashimoto, Mami Tanaka, Naoki Saito, Mamiko Endo, Jun Okayama, Akiko Ichihara, Saki Eshima, Satoshi Handa, Masayoshi Senda, Yasunori Sato, Hiroyuki Watanabe, Michiko Nakazato, Masaomi Iyo

**Affiliations:** 1 Department of Psychiatry, Graduate School of Medicine, Chiba University, 1-8-1 Inohana, Chuou-ku, Chiba, Chiba 260-8670 Japan; 2 Department of Psychiatry, Sodegaura Satsukidai Hospital, 5-21 Nagauraekimae, Sodegaura, Chiba 299-0246 Japan; 3 Division of Clinical Study on Juvenile Delinquency, Center for Forensic Mental Health, Chiba University, 1-8-1 Inohana, Chuou-ku, Chiba, Chiba 260-8670 Japan; 4 Department of Pediatrics, Graduate School of Medicine, Chiba University, 1-8-1 Inohana, Chuou-ku, Chiba, Chiba 260-8670 Japan; 5 Division of Clinical Forensic Medicine, Education and Research Center of Legal Medicine, Chiba University, 1-8-1 Inohana, Chuou-ku, Chiba, Chiba 260-8670 Japan; 6 Department of Reproductive Medicine, Graduate School of Medicine, Chiba University, 1-8-1 Inohana, Chuou-ku, Chiba, Chiba 260-8670 Japan; 7 Welfare and Medical Intelligence, Chiba University Hospital, 1-8-1 Inohana, Chuou-ku, Chiba, Chiba 260-8677 Japan; 8 Department of Psychiatry, School of Medicine, International University of Health and Welfare, 4-3 Kozunomori, Narita, Chiba 286-8686 Japan; 9 Department of Pediatrics, Asahi General Hospital, I-1326, Asahi, Chiba 289-2511 Japan; 10 Department of Preventive Medicine and Public Health, Keio University School of Medicine, 35 Shinanomachi, Shinju-ku, Tokyo 160-8582 Japan; 11 Division of Medical Treatment and Rehabilitation, Center for Forensic Mental Health, Chiba University, 1-8-1 Inohana, Chuou-ku, Chiba, Chiba 260-8677 Japan; 12 Gakuji-Kai Kimura Hospital, 6-19, Higashi-Honcho, Chuou-ku, Chiba, Chiba 260-0004 Japan

**Keywords:** Child abuse, Child maltreatment, Factor, Inter-agency collaboration, Multidisciplinary approach, Prevention of child maltreatment

## Abstract

**Objectives:**

This study aimed to clarify the factors of successful inter-agency collaboration that affect multidisciplinary workers’ abilities to identify child maltreatment. A questionnaire-based survey was conducted; the contents of the questionnaire included the Collaboration Evaluation Scale we developed and the workers’ abilities to identify child maltreatment. In total, 277 individuals from various agencies in Japan participated in this study. To examine the factors of successful inter-agency collaboration affecting workers’ awareness of child maltreatment, we used hierarchical multiple regression analysis.

**Results:**

The analysis showed the positive effect of “commitment with loyalty” on the workers’ awareness of child maltreatment-related information in all fields (*β* = .18–.31, *p* < .05), the effect of “strong leadership” on information about maltreated children and the home environment (*β* = .18, *p* < .05; *β* = .16, *p* < .05, respectively), and the effect of “resources” on the information about mothers’ information during pregnancy and of fathers’ feelings towards their children during the perinatal period (*β* = .17, *p* < .05; *β* = .22, *p* < .01, respectively). In conclusion, commitment with loyalty, strong leadership, and resources are factors of successful inter-agency collaboration that affects the ability of multidisciplinary workers to recognize signs of child maltreatment.

## Introduction

Child maltreatment is an extremely serious issue worldwide [1]. In Japan, the number of reported cases of child maltreatment was 133,778 in 2017, presenting a threefold increase from the previous decade [2]. Child maltreatment has negative consequences, such as physical injuries that can result in death; impairments of physical, socioemotional, and cognitive development; and various mental health problems, including not only diagnosable diseases but also serious behavioral issues [3–5].

A multidisciplinary approach from multi-agency partnerships is a standard in the prevention of child maltreatment [6, 7]. Given that early interventions, such as those conducted by nurses visiting new parents and pregnant women at risk of child maltreatment, are effective methods for preventing child maltreatment [8], it is important that multidisciplinary workers who belong to the agencies involved with families and pregnant women at risk of child maltreatment are aware of the early signs of child maltreatment [5, 9]. Therefore, the evaluation and improvement of the ability of such workers’ awareness of the signs of child maltreatment is needed in inter-agency collaboration practices.

In Japan, child protection services (CPS), in which child guidance centers located in each administrative jurisdiction play a central role, deal with suspected child maltreatment cases. To prevent child maltreatment, the CPS collaborates with various agencies, such as hospitals, schools, educational boards, municipal offices, police, and public health centers. However, there are many issues regarding functional practices in multidisciplinary collaboration among agencies. Several studies have discussed the barriers to, and factors for, successful collaboration in child protection [10–12]. Johnson et al. investigated the seven factors of successful inter-agency collaboration: (1) commitment, (2) communication, (3) strong leadership provided by key decision-makers, (4) an understanding of the collaborating agencies’ cultures, (5) engagement in serious preplanning, (6) provision of adequate resources for collaboration, and (7) turf issues [12].

We hypothesize that the above-mentioned factors of successful inter-agency collaboration could affect the ability of workers involved with families and pregnant women at risk of child maltreatment to recognize signs of child maltreatment. This study aims to clarify the effects of these factors of successful inter-agency collaboration on multidisciplinary workers’ abilities to recognize signs of child maltreatment. Specifically, the following research questions were examined: (1) What are the pivotal factors of successful inter-agency collaboration?; (2) Which of these factors affect the ability of workers involved with children, families, and pregnant women at risk of child maltreatment to recognize signs of child maltreatment?

## Main text

### Methods

#### Participants and data collection

The survey was conducted based on the data collected from August to September 2018. Participants were anonymous multidisciplinary workers who were involved with children and families at risk of child maltreatment. The questionnaire sheet was mailed to the following institutions: 36 hospitals with pediatric emergency departments; 119 child protection-related departments of all municipal offices in Chiba Prefecture, such as the department of children’s health, family care, and prenatal care; seven child guidance centers; 19 public health centers; 54 local boards of education; and one district public prosecutor’s office in Chiba Prefecture (i.e. 236 facilities in total).

#### Measures

##### Collaboration evaluation scale

We developed a Collaboration Evaluation Scale based on the seven factors related to successful inter-agency collaboration regarding Johnson’s study [12]. It contained 24 items, and participants rated each item using a four-point Likert scale ranging from 1 (strongly disagree) to 4 (strongly agree).

##### Assessment of workers’ abilities to recognize child maltreatment

We developed the questionnaire sheet with reference to the previously reported risk factors of child maltreatment [13, 14]. The questionnaire consisted of 41 items related to maltreatment to assess responders’ abilities to recognize signs of child maltreatment (see Additional file 1). Participants rated each item using a six-point Likert scale, ranging from 1 (not important at all) to 6 (very important).

#### Data analysis

Statistical analysis was performed using the SPSS 19.0J software package (SPSS, Inc., Chicago, IL). Before performing hierarchical multiple regression analysis to reveal the factors of successful inter-agency collaboration affecting multidisciplinary workers’ awareness of child maltreatment, data screening was conducted to confirm that the assumptions of multivariate analysis were satisfied. We used Cronbach’s *α* to analyze the internal consistency of the Collaboration evaluation scale and the questions for information to assess the risk of child maltreatment. After that, we calculated the correlations between factors of the Collaboration Evaluation scale and categories of items of information for assessing the risk of child maltreatment and then conducted a hierarchical multiple regression analysis of factors of the Collaboration Evaluation scale and the categories of items for assessing the risk of child maltreatment. For all statistical tests, we used a significance level of .05.

## Results

The completed questionnaires were returned by 277 individuals from 114 facilities (48.3%). A total of 277 individuals, comprising 67 men, 207 women, and 3 individuals who did not report their sex, participated in this study. Participants ranged from 22 to 68 years old (mean = 43.6, standard deviation (SD) = 10.8). Table 1 displays the participants’ sex, age, agency, profession, and years of child protection experience.

**Table 1:** Social and Demographic Characteristics

	*n*	%
Sex		
Male	67	24.2
Female	207	74.7
Unknown	3	1.1
Age (years)		
< 30	31	11.2
31–40	65	23.5
41–50	91	32.9
51–60	65	23.5
> 60	20	7.2
Unknown	5	1.8
Years of child protection experience		
< 1 year	27	9.7
1 < 2 years	33	11.9
2 < 4 years	60	21.7
4 < 6 years	27	9.7
6 < 8 years	23	8.3
8 < 10 years	14	5.1
≥ 10 years	81	29.2
Unknown	12	4.3
Agency		
Child guidance centre	15	5.4
Hospital	59	21.3
State municipality	66	23.8
Public health centre	58	20.9
Prosecutors	2	.7
Board of education	30	10.8
Child and family support centre	30	10.8
Others	17	6.1
Profession		
Child welfare officer	14	5.1
Physician	24	8.7
Nurse	19	6.9
Other healthcare professional	1	.4
Clinical psychologist	7	2.5
Medical social worker	21	7.6
Public health nurse	96	34.7
Legal professional	2	.7
Teaching professional	17	6.1
Welfare officer for child and family	43	15.5
Others	33	11.9

Sample size, *n* = 277

Table 2 shows that the result of the exploratory factor analysis (EFA) on the Collaboration Evaluation Scale. We found four factors as a result of EFA of the 24 item: “commitment with loyalty” (10 items; *α* = .84), “strong leadership” (6 items; *α* = .87), “resources” (4 items; *α* = .62), and “turf issues” (4 items; *α* = .63).

**Table 2:** Factor loadings of the items of the collaboration evaluation scale

Item	(*n* = 277)			
	1	2	3	4
Factor 1^a^: Commitment and Loyalty (*α* = .84)				
1. I take time to learn and understand each collaborating agency’s mission and priorities	.71			
2. I always keep the goals and the potential positive outcomes of the collaboration in mind	.69			
3. The characteristics of each collaborating agency are utilised to the maximum	.64			
4. Prior to the beginning of a new collaboration, I identify similarities/differences between the cultures of the participating agencies	.60			
5. I develop a way to compromise on important differences	.59			
6. I provide feedback on the results of the cases to the collaborating agency	.58			
7. I think the establishment of trust and mutual responsibility for common goals is important for collaborating with other agencies	.55			
8. I define the goal of the case in detail prior to beginning a new collaboration	.49			
9. I understand the working process of the collaborating agency	.48			
10. I talk to my colleagues about a positive view of the collaboration	.42			
Factor 2: Strong Leadership (*α* = .87)				
11. The upper management can take responsibility for making decisions on behalf of the collaborating agency		.89		
12. The upper management provides immediate assistance when problems arise		.74		
13. The upper management truly understands our agency’s position in the collaboration		.73		
14. The upper management truly understands the priorities of the collaboration		.69		
15. The upper management has a positive attitude towards the utilisation of our agency’s resources to support the collaboration		.69		
16. The upper management explains the role of our agency to other collaborating agencies		.53		
Factor 3: Resources (*α* = .62)				
17. I review pertinent laws and regulations prior to the collaborative effort			.55	
18. I check the similar issues and cases of previous inter-agency collaborations prior to beginning a new collaboration			.52	
19. There are sufficient staff members to engage in the collaboration			.49	
20. There are sufficient funding sources to engage in the collaboration			.39	
Factor 4: Turf Issues (*α* = .63)				
21. I do not want to deal with the additional tasks pertaining to the collaboration				.72
22. I do not want to provide any additional resources for the collaborating agency when my agency’s duty is finished				.61
23. I want to defend my own territory				.46
24. I do not want to examine or modify an agency’s procedures that are unnecessarily inhibiting or detrimental to a collaborating agency				.44

^a^An exploratory factor analysis was performed, resulting in a four-factor solution (which explains 42.0% of the variance)

Regarding the assessment of workers’ abilities to recognize child maltreatment, we categorized 41 items into seven fields and performed principal component analysis for each field: (1) maltreated children (10 items, *α* = .90), including whether they had any physical injuries, their developmental state, and the degree of their affection towards their guardians; (2) mothers’ information, including their child-rearing ability (9 items, *α* = .91), such as the mother’s age, intellectual ability, and mental disorder; (3) mothers’ information during pregnancy (4 items, *α* = .88), including whether the mother was socially isolated during pregnancy and whether the pregnancy was unexpected; (4) mothers’ information after childbirth (6 items, *α* = .91), including whether the mother was socially isolated after childbirth and the mother’s feelings towards her child after childbirth; (5) fathers’ information, including their child-rearing ability (5 items, *α* = .85), such as the father’s age, intellectual ability, and mental disorder; (6) fathers’ feelings towards his children during pregnancy and after childbirth (2 items, *α* = .94), including what types of feelings the father had towards his child during pregnancy and after childbirth; and (7) the home environment (5 items, *α* = .88), including each home’s economic situation, whether there was a supporter, and whether there was a record of child maltreatment.

We analyzed the correlations between the Collaboration Evaluation Scale’s four factors and the seven categories of items of information for assessing the risk of child maltreatment. Commitment with loyalty factor had a significant positive correlation with all the seven categories (*r* = .23–.40, *p* < .01). Further, the strong leadership factor was significantly positively correlated with mothers’ information, including their child-rearing ability (*r* = .21, *p* < .01); fathers’ information, including their child-rearing ability (*r* = .13, *p* < .05); the home environment (*r* = .24, *p* < .01); and maltreated children (*r* = .22, *p* < .01). Resources were significantly positively correlated with mothers’ information during pregnancy and fathers’ feelings towards their children during pregnancy and after childbirth (*r* = .19, *p* < .01; *r* = .24, *p* < .01, respectively).

A hierarchical multiple regression analysis was conducted using the four factors of the Collaboration Evaluation scale and the scores of the seven categories of items for assessing the risk of child maltreatment as objective variables. In Step 1, we entered the years of child protection experience, sex, and age as basic attributes, and in Step 2, we entered the scale’s four factors. The forced entry method was adopted to perform all the steps. Significant differences were found in the increase in the coefficient of determination between the steps. In the second model, which was the final step, the value of the partial regression coefficient (*β*) for each variable indicated commitment with loyalty, strong leadership, resources, and turf issues. There were positive influences of (1) commitment with loyalty on all the categories, (2) strong leadership on maltreated children and the home environment, and (3) recourses on mothers’ information during pregnancy and fathers’ feelings towards their children during pregnancy and after childbirth (Table 3).

**Table 3:** Hierarchical regression models explaining the multidisciplinary workers’ ability to identify child maltreatment

Step	Factor	Maltreated children^a^	Mothers’ basic information including their child-rearing ability^b^	Mothers’ information during pregnancy^c^	Mothers’ information after childbirth^d^	Fathers’ basic information including their child-rearing ability^e^	Fathers’ feelings towards their children during pregnancy and after childbirth^f^	Home environment^g^
		*β*	*β*	*β*	*β*	*β*	*β*	*β*
1	Age	− .01	.00	− .01	− .38	.05	.06	.06
	Sex	.20^***^	.33^***^	.30^***^	.34^***^	.23^***^	.19^**^	.22^***^
	Years of child protection experience	.28^***^	.19^**^	.16^*^	.18^**^	.18^**^	.02	.16^**^
2	Commitment with loyalty	.30^***^	.30^***^	.18^*^	.25^***^	.28^***^	.21^**^	.31^***^
	Strong leadership	.18^*^	.13	.01	.03	.02	− .11	.16^*^
	Resources	− .08	− .04	.17^*^	.02	.01	.22^**^	− .03
	Turf issues	− .02	− .04	− .03	− .01	− .04	− .03	− .07
	Step 1 *R*^*2*^	.12^***^	.15^***^	.11^***^	.15^***^	.10^***^	.05^*^	.09^***^
	Step 2 *R*^*2*^	.26^***^	.28^***^	.20^***^	.23^***^	.19^***^	.14^***^	.25^***^

The contents of each category are as follows

^a^Whether they have any physical injuries, developmental state, the degree of their affection towards their guardians, and so on

^b^Mother’s age, intellectual ability, mental disorder, and so on

^c^Whether or not the mother was socially isolated during pregnancy, whether or not the pregnancy was unexpected, and so on

^d^Whether or not the mother was socially isolated after childbirth, the mother’s feelings towards her child after childbirth, and so on

^e^The father’s age, intellectual ability, mental disorder, and so on

^f^What kinds of feelings the father had towards his child during pregnancy and after childbirth

^g^The economic situation of each home, whether there is a supporter or not, whether there is a record of child abuse, and so on

* *p *< .05, ** *p *< .01, *** *p* < .001

## Discussion

We determined that (i) commitment with loyalty, (ii) strong leadership, (iii) resources, and (iv) turf issues were key factors for inter-agency collaboration among multidisciplinary workers involved with children, families, and pregnant women at risk of child maltreatment. Our results show how these four factors affect workers’ ability to recognize signs of child maltreatment.

This study showed that the commitment with loyalty factor had a positive influence on all the areas of information that workers considered important signs of child maltreatment. This supports the idea that commitment factor is a basic, essential, and pivotal element in the provision of children and family support services through inter-agency collaboration [15, 16].

We found that strong leadership also is a factor of successful inter-agency collaboration affecting multidisciplinary workers’ ability to identify “maltreated children” and “home environment problems” in addition to commitment with loyalty. This finding is consistent with that of previous studies on the provision of services for maltreated or disabled children [12, 15, 17]. If upper management does not provide leadership, the efficiency of inter-agency collaboration will decrease [18]. Our study provides a new finding that strong leadership affects workers’ awareness of “maltreated children” and “home environment problems.” To improve workers’ awareness of signs of child maltreatment, people who excel in management and leadership should be assigned to each agency involved in child maltreatment, such as schools, nursery schools, child guidance centers, and municipal offices.

The present study demonstrates that resources affect multidisciplinary workers’ abilities to respond to “mothers’ information during pregnancy” and “fathers’ feelings towards their children during pregnancy and after childbirth,” in addition to commitment with loyalty. Much previous research has emphasized the importance of allocating sufficient resources for inter-agency collaboration within CPS [19, 20]. Johnson et al. found that stable funding is vital and individual staff members should not engage in inter-agency collaboration without funding [12]. Fifty percent of women with postpartum depression suffer from depression during pregnancy [21]. The Edinburgh Postnatal Depression Scale’s scores are significantly positively correlated with neglectful or aggressive parenting behavior of mothers with a child below 1 year old [22]. Further, fathers’ positive attitudes towards their children can reduce the maternal risk for physical child abuse [23]. The improvement of resources for inter-agency collaboration to prevent child maltreatment may improve multidisciplinary workers’ abilities to identify pregnant women and their partners who are at risk of child maltreatment before birth. Further studies are required to clarify the relationships between relevant resources.

The results showed that turf issues as a factor of inter-agency collaboration do not affect workers’ ability to recognize child maltreatment. Since many reports mention that turf issues or the division of roles hinder good inter-agency collaboration [12, 24], these results were unexpected. One potential reason is that a bias in the recruitment of participants may have influenced the results. Only those who were interested in this study’s purpose responded to the questions. Those who did not respond might have been inactive or uncooperative.

## Conclusion

This study identified three factors—commitment with loyalty, strong leadership, and resources—as successful inter-agency collaboration practices to prevent child maltreatment. Furthermore, these factors affect the ability of multidisciplinary workers to recognize signs of child maltreatment. To improve abilities to prevent child maltreatment in inter-agency collaboration practices, the present findings could be useful.

## Limitations

This study has several limitations. First, as participants could choose whether or not to answer the survey questions, their answers may have been biased since only those who were interested in the study’s purpose responded to the questions. Second, regional characteristics may have affected the answers, since our survey was only conducted in Chiba Prefecture in the Tokyo Metropolitan Area. However, Chiba Prefecture, which is inhabited by more than 600 million people, has a balanced number of rural and urban regions. Finally, we did not enquire about the details of cases handled by individual participants, such as the seriousness of the abuse, which would require the separation of children from their parents. Further research that overcomes the above problems should be conducted on this topic.

## Supplementary information

**Additional file 1.** The self-assessment sheet for workers’ abilities to recognize child maltreatment.

## Data Availability

The datasets supporting the conclusions drawn in this article are available upon reasonable request.

## Abbreviations

CPS: Child protection services
CGCs: Child guidance centers
EFA: Exploratory factor analysis
