# Supplementary material for: Inter-agency collaboration factors affecting multidisciplinary workers’ ability to identify child maltreatment
Source: BMC Res Notes. 2020 Jul 6;13:323. doi: 10.1186/s13104-020-05162-7 (PMC7339586; doi:10.1186/s13104-020-05162-7)
Supplement: Supplementary file 1 — Additional file 1. The self-assessment sheet for workers’ abilities to recognize child maltreatment. [file 13104_2020_5162_MOESM1_ESM.docx]

Inter-Agency Collaboration to Address Child Maltreatment and Worker Awareness of Factors Predicting Maltreatment

C）Please rate the importance of each of these factors for you when you are addressing a potential case of child maltreatment by circling the number of the appropriate response.

|  |  | Not important at all | Not very important | Somewhat unimportant | Somewhat important | Moderately important | Very important |
| --- | --- | --- | --- | --- | --- | --- | --- |
|  | The child had health problems at birth (e.g., low birth weight, congenital diseases or disorders) | 1 | 2 | 3 | 4 | 5 | 6 |
|  | The child has developmental problems (e.g., poor growth or weight gain, delayed speech) | 1 | 2 | 3 | 4 | 5 | 6 |
|  | The child has inexplicable injuries or bruises | 1 | 2 | 3 | 4 | 5 | 6 |
|  | The child exhibits problematic behaviors | 1 | 2 | 3 | 4 | 5 | 6 |
|  | The way how the child acts toward parents/caregivers | 1 | 2 | 3 | 4 | 5 | 6 |
|  | Mother’s age at pregnancy/childbirth | 1 | 2 | 3 | 4 | 5 | 6 |
|  | Father’s age at pregnancy/childbirth | 1 | 2 | 3 | 4 | 5 | 6 |
|  | Mother’s physical health | 1 | 2 | 3 | 4 | 5 | 6 |
|  | Mother’s mental health | 1 | 2 | 3 | 4 | 5 | 6 |
|  | Father’s physical health | 1 | 2 | 3 | 4 | 5 | 6 |
|  | Father’s mental health | 1 | 2 | 3 | 4 | 5 | 6 |
|  | Mother’s intellectual abilities | 1 | 2 | 3 | 4 | 5 | 6 |
|  | Father’s intellectual abilities | 1 | 2 | 3 | 4 | 5 | 6 |
|  | Maternal developmental disabilities | 1 | 2 | 3 | 4 | 5 | 6 |
|  | Paternal developmental disabilities | 1 | 2 | 3 | 4 | 5 | 6 |
|  | Mother’s parenting skills | 1 | 2 | 3 | 4 | 5 | 6 |
|  | Problems related to mother’s personality (e.g., irritability, impulsivity, emotional instability, egotism, lack of empathy) | 1 | 2 | 3 | 4 | 5 | 6 |
|  | Mother’s feelings toward the child during pregnancy | 1 | 2 | 3 | 4 | 5 | 6 |
|  | Mother’s feelings toward the child after birth | 1 | 2 | 3 | 4 | 5 | 6 |
|  | Father’s feelings toward the child during his wife’s pregnancy | 1 | 2 | 3 | 4 | 5 | 6 |
|  | Father’s feelings toward the child after birth | 1 | 2 | 3 | 4 | 5 | 6 |
|  | Mother’s history with her own parents (e.g., abuse history, parental conflicts) | 1 | 2 | 3 | 4 | 5 | 6 |
|  | Substance use during pregnancy (e.g., tobacco, alcohol) | 1 | 2 | 3 | 4 | 5 | 6 |
|  | Substance use after childbirth (e.g., tobacco, alcohol) | 1 | 2 | 3 | 4 | 5 | 6 |
|  | Mother’s postpartum health (e.g., postpartum depression, increase in stress) | 1 | 2 | 3 | 4 | 5 | 6 |
|  | Maintenance of regular well-baby checkups and immunizations | 1 | 2 | 3 | 4 | 5 | 6 |
|  | Marital situation after childbirth (e.g., single mother) | 1 | 2 | 3 | 4 | 5 | 6 |
|  | Change in family structure (e.g., divorce, death, or remarriage of a parent, and whether the step-parent brought children with them) | 1 | 2 | 3 | 4 | 5 | 6 |
|  | Child’s birth order in the family (e.g., Are there siblings? How many? Does someone have physical or psychological disorders?) | 1 | 2 | 3 | 4 | 5 | 6 |
|  | Child’s home environment (e.g., homes with a single parent (sometimes never-married), a common-law spouse or another live-in person, remarried parents with children from previous marriages) | 1 | 2 | 3 | 4 | 5 | 6 |
|  | Good marital relationship | 1 | 2 | 3 | 4 | 5 | 6 |
|  | The way how the child acts toward others (e.g., is the child aggressive or over-familiar with strangers） | 1 | 2 | 3 | 4 | 5 | 6 |
|  | Whether the mother was isolated during pregnancy from other family members or her local community | 1 | 2 | 3 | 4 | 5 | 6 |
|  | Whether the mother was isolated after childbirth from other family members or her local community | 1 | 2 | 3 | 4 | 5 | 6 |
|  | Mother’s relationships with others during pregnancy (e.g., did she have people to discuss things with or did she have troubled relationships with others) | 1 | 2 | 3 | 4 | 5 | 6 |
|  | Mother’s relationships with others after giving birth (e.g., did she have people to discuss things with or did she have troubled relationships) | 1 | 2 | 3 | 4 | 5 | 6 |
|  | Family’s financial situation | 1 | 2 | 3 | 4 | 5 | 6 |
|  | Whether the child has any physical, intellectual or developmental disabilities | 1 | 2 | 3 | 4 | 5 | 6 |
|  | Whether the principal caregiver has support from people other than their partner | 1 | 2 | 3 | 4 | 5 | 6 |
|  | Is the child going to daycare, kindergarten, or elementary school? | 1 | 2 | 3 | 4 | 5 | 6 |
|  | Whether the family has a history of reported abuse | 1 | 2 | 3 | 4 | 5 | 6 |
